# The incidence, aetiology and outcome of acute seizures in children admitted to a rural Kenyan district hospital

**DOI:** 10.1186/1471-2431-8-5

**Published:** 2008-02-08

**Authors:** Richard Idro, Samson Gwer, Michael Kahindi, Hellen Gatakaa, Tony Kazungu, Moses Ndiritu, Kathryn Maitland, Brian GR Neville, Piet A Kager, Charles RJC Newton

**Affiliations:** 1 Centre for Geographic Medicine Research – Coast, Kenya Medical Research Institute, Kilifi, Kenya; 2 Department of Paediatrics and Child Health, Mulago Hospital/Makerere University, Kampala, Uganda; 3 Department of Paediatrics, Faculty of Medicine and The Wellcome Trust Centre for Tropical Medicine, Imperial College, London, UK; 4 Neurosciences Unit, The Wolfson Centre, University College London, Institute of Child Health, UK; 5 Department of Infectious Diseases, Tropical Medicine and AIDS, Academic Medical Centre, Amsterdam, The Netherlands

## Abstract

**Background:**

Acute seizures are a common cause of paediatric admissions to hospitals in resource poor countries and a risk factor for neurological and cognitive impairment and epilepsy. We determined the incidence, aetiological factors and the immediate outcome of seizures in a rural malaria endemic area in coastal Kenya.

**Methods:**

We recruited all children with and without seizures, aged 0–13 years and admitted to Kilifi District hospital over 2 years from 1^st ^December 2004 to 30^th ^November 2006. Only incident admissions from a defined area were included. Patients with epilepsy were excluded. The population denominator, the number of children in the community on 30^th ^November 2005 (study midpoint), was modelled from a census data.

**Results:**

Seizures were reported in 900/4,921(18.3%) incident admissions and at least 98 had status epilepticus. The incidence of acute seizures in children 0–13 years was 425 (95%CI 386, 466) per 100,000/year and was 879 (95%CI 795, 968) per 100,000/year in children <5 years. This incidence data may however be an underestimate of the true incidence in the community. Over 80% of the seizures were associated with infections. Neonatal infections (28/43 [65.1%]) and falciparum malaria (476/821 [58.0%]) were the main diseases associated with seizures in neonates and in children six months or older respectively. Falciparum malaria was also the main illness (56/98 [57.1%]) associated with status epilepticus. Other illnesses associated with seizures included pyogenic meningitis, respiratory tract infections and gastroenteritis. Twenty-eight children (3.1%) with seizures died and 11 surviving children (1.3%) had gross neurological deficits on discharge. Status epilepticus, focal seizures, coma, metabolic acidosis, bacteraemia, and pyogenic meningitis were independently associated with mortality; while status epilepticus, hypoxic ischaemic encephalopathy and pyogenic meningitis were independently associated with neurological deficits on discharge.

**Conclusion:**

There is a high incidence of acute seizures in children living in this malaria endemic area of Kenya. The most important causes are diseases that are preventable with available public health programs.

## Background

Acute seizures are a common neurological symptom in sick children. In patients with fever, they include febrile seizures [1,2], acute symptomatic seizures (e.g. in a child with pyogenic meningitis)[3] or initial seizures in a child with epilepsy or epilepsy syndrome[2].

Worldwide, febrile seizures are the most common type of acute seizures in children[4]. Most are associated with infections and have a good outcome[5]. In tropical countries, febrile seizures are common but the prevalence of acute symptomatic seizures (which have a poorer outcome) may be higher than Western countries [6-8]. The incidence of both acute seizures and febrile status epilepticus is higher[2,9] and the outcome is worse since the aetiology is different[6,8,10,11]. Acute seizures are therefore a major risk factor for neurological and cognitive impairment [12-14] and for the development of epilepsy [15-17] in children living in these regions. However few studies have examined the burden and risk factors for acute seizures in sub-Saharan Africa. Most of the available reports are of rates in hospital in the 1990's and are limited to the diagnoses associated with the seizure-events [18-20].

In this study, we recruited all children with incident acute seizures from a defined area in coastal Kenya admitted to a district hospital to determine the incidence, the aetiology and outcome of the seizures.

## Methods

### Study participants

Participants were children who were admitted with a history of acute convulsions, to Kilifi District Hospital over a period of 2 years from 1^st ^December 2004 to 30^th ^November 2006 and who were resident in a defined area in coastal Kenya. Children from the same study area but admitted without a history of seizures formed the comparison group. This hospital admits about 5000 children <14 years annually and is the only hospital in the area that admits very sick children. The study area undergoes a regular census three times per year and is defined by an area where over 60% of patients attending our hospital live. Each person in the study area has a unique identifier and data from hospital admissions is individually linked to the census data. However, not every child with a seizure from this community is attended to in hospital. From previous studies, just over 20% of children who had seizures in the community were admitted to the hospital (Anthony Ngugi, personal communication). The annual entomological inoculation rate (the number of times an individual is bitten by mosquitoes infected with *Plasmodium falciparum *in one year) in the catchment area ranges from <1 to 120 infectious bites per year[21].

### Admission procedures

Ethical approval for the study was granted by the Kenya Medical Research Institute. Study participants were recruited consecutively as they presented to hospital. At admission, emergency care and resuscitation procedures such as correction of hypoglycaemia, hypovolaemia, oxygen and anticonvulsant therapy were administered according to Kenyan guidelines. Parents were then invited to consent to participate in the study. Children with epilepsy (two or more lifetime episodes of unprovoked seizures) were excluded. The history included the number and a parental description of the seizures. Level of consciousness was determined using the Blantyre Coma Scale [22]. Status epilepticus was defined as a seizure lasting 30 minutes or longer or as three or more seizures from which the patient did not regain consciousness after one hour[23].

About 5 ml of venous blood was obtained for a full blood count, blood glucose, malaria parasites, microbiological culture, and plasma from children with life threatening features (acidotic breathing, shock, prostration or impaired consciousness) was obtained for acid base status, electrolytes and creatinine. The volume of blood drawn depended on the weight of the child and the ability to tolerate the blood draw. Thus, only 2 ml was drawn from very low birth weight infants. A growth of micrococci, coagulase negative staphylococci or gram-positive Bacillus species was considered a contaminant. Severe anaemia was defined as hemoglobin <50 g/L and metabolic acidosis as a base deficit >8. Hypokalaemia was defined as plasma K^+^<3.0 mmol/L, hyperkalaemia as K^+^>5.0 mmol/L, hyponatraemia (moderate-severe) as Na^+^<125 mmol/L and impaired renal function as plasma creatinine >80 μmol/L. Lumbar punctures were performed according to a standard protocol to exclude pyogenic meningitis[24]. In summary, all admissions of children aged <60 days with suspected sepsis, all those with impaired consciousness, children ages <6 months or >6 years with any convulsions or children with history of partial, prolonged or atypical seizures (i.e. not a simple febrile convulsion), signs of meningitis, suspected tuberculous meningitis, cryptococcal disease, acute flaccid paralysis, encephalitis or those being evaluated for CNS involvement in malignancies had lumbar puncture.

A child was said to have malaria as a primary diagnosis if s/he had asexual forms of P. falciparum parasites detected on blood films and malaria was the only or main diagnosis. Children with malaria parasitaemia but admitted because of other clinical conditions (e.g. acute trauma, cardiovascular disease) were deemed to have other primary diagnoses. Malaria was treated with parenteral quinine (a loading dose of 15 mg/kg in 5% dextrose then 10 mg/kg 12 hourly until the child could take orally when antimalarial treatment was completed with a full course of Artemether-lumefantrine). Bacterial infections in children ≤ 2 months were treated with a combination of Gentamicin (a loading dose of 4 mg/kg and a continuation dose of 2 mg/kg every 24 hours for babies <2 kg or a loading dose of 7 mg/kg and a continuation dose of 4 mg/kg every 24 hours for babies ≥ 2 kg) and Ampicillin (50 mg/kg/6 hrly). For older children, crystalline penicillin (60 mg/kg/6 hourly) and Chloramphenicol (loading dose 40 mg/kg and a continuation dose of 25 mg/kg/6 hourly) was used. Using the above regimes, children with pyogenic meningitis received 14 days of antibiotics. Neonatal gram negative meningitis was treated for 21 days. Where resistance to the first line antibiotics was demonstrated, Ceftriaxone (or other appropriate antibiotic) was administered as 100 mg/kg/day in 2 divided doses. There were no facilities to identify viral causes of meningo-encephalitis and acyclovir was unavailable.

### Data management and analysis

Individual patient clinical data was directly entered in a FileMaker 5.5 database at admission. Data was analysed using Stata version 9.2 (Stata Corporation, Texas). Using the unique patient identification numbers, the first admission during the two-year period was defined as the incident admission. The denominator, the population of children 0–13 years was 105,992 and was obtained by modelling the census data to the date of 30 November 2005 (the midpoint of the study period). This age and sex specific estimate of the population denominator was obtained by fitting a linear regression line through the census counts (on a log scale) of October 2005 and February 2006. The incidence rates of acute seizures and status epilepticus are expressed as events per 100,000/year. A map was constructed by plotting the incidence by area of patient residence. In addition, we plotted the monthly admissions with acute seizures and compared this with the number of admissions with malaria. We then compared the clinical and laboratory features and the diagnoses in patients with and without seizures to describe the clinical risk factors for seizures and examined the risk factors for poor outcome. A poor outcome was defined as death or the presence of a gross neurological deficit/sequela on discharge. Normally distributed continuous data were compared using the unpaired Student's t-test while skewed data were compared using Mann Whitney-Wilcoxon's rank-sum test. Pearson's chi square test (or Fischer's exact test as appropriate) was used to compare proportions. Results are presented with crude odds ratios and a p value < 0.05 is considered significant. To determine risk factors independently associated with mortality or neurological deficits, clinical and laboratory parameters with a p value < 0.1 at univariate analysis were entered in a logistic regression model using a stepwise entry system.

## Results

### General description

**Figure 1:**
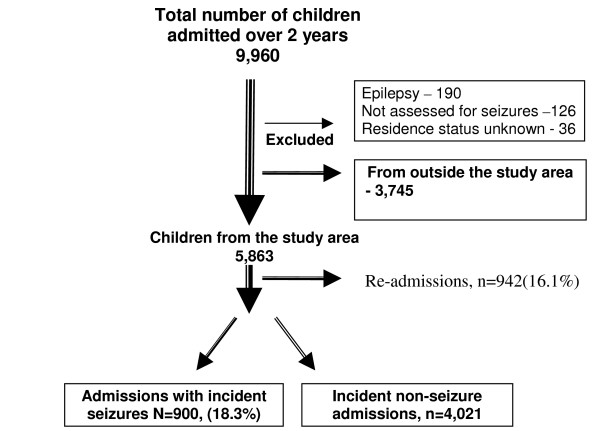
Admissions to KDH, December 2004 to November 2006.

A total of 9,960 children aged 0–13 years were admitted to KDH during the 2-year period. We excluded the 3,745 children from outside the study area from the analysis. In addition, 190 children with epilepsy, 36 whose residence status in the DSS could not be ascertained and 126 children not assessed for a history of seizures were excluded (figure 1).

### The incidence of acute seizures and status epilepticus in Kilifi DSS

**Figure 2:**
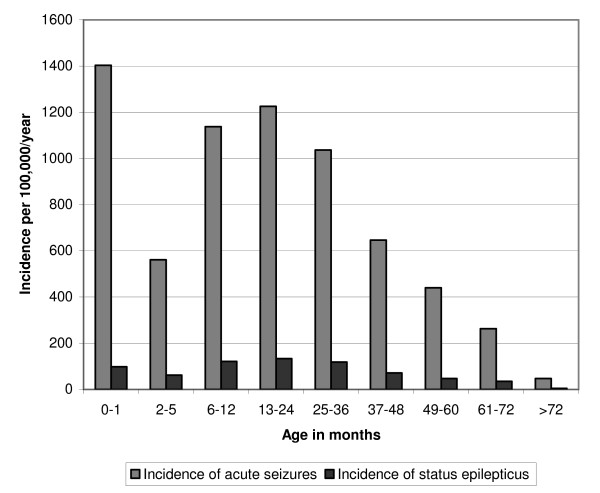
The incidence of acute seizures and status epilepticus by age group in children in Kilifi study area.

Among the remaining 5,863 admissions from the study area, there were 942(16.1%) children defined as re-admissions. These non-incident admissions were excluded from further analysis. Nine hundred (18.3%) out of the 4,921 incident admissions had seizures. The incidence of acute seizures in children 0–13 years was 425 (95% CI 386, 466) per 100,000/year and was 879 (95% CI 795, 968) per 100,000/year in children <5 years. The incidence was highest (1,403 [95%CI 902, 2166] per 100,000/year) in neonates, lowest in infants aged 2–5 months with a second peak of 1,226 (95% CI 1008, 1476) per 100,000/year in children 13–24 months (figure 2). At least 98(10.9%) children with seizures had status epilepticus. The overall incidence rate of status epilepticus was 46 (95% CI 32, 58) per 100,000/year and was 95 (95% CI 69, 128) per 100,000/year in children <5 years.

**Figure 3:**
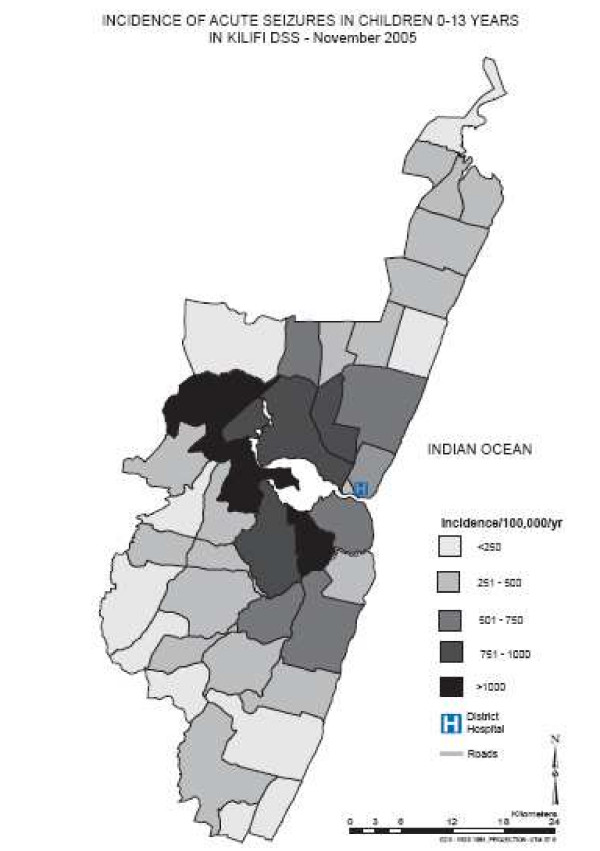
**Incidence of acute seizures in Kilifi study area**. Figure 3 is a map of Kilifi demographic surveillance area and it shows the incidence for acute seizures in the study area by sub-location. In general, the incidence is highest in areas nearest to the district hospital and decreases with distance away from the hospital.

**Figure 4:**
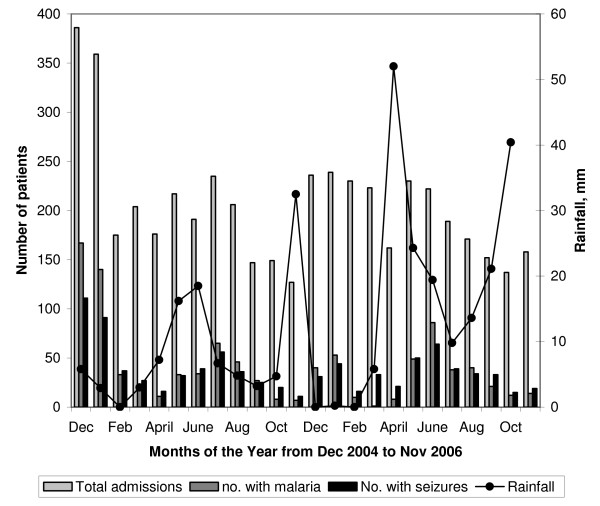
**Monthly admissions with malaria, seizures and rainfall in Kilifi**. Total monthly admissions, admissions with malaria and seizures, and rainfall: The number of patients admitted with seizures has a seasonal pattern with peaks in December-January (after the short rains) and May-August (after the long rains). These peaks coincide with that for patients with presenting with malaria.

In general, zones nearest to the hospital had higher incidence rates of acute seizures (figure 3). Children with seizures were admitted throughout the year, with increases in May-August and December-January, the months that followed the long and short rains respectively (figure 4).

### Risk factors for acute seizures and aetiological diagnoses associated with seizures

#### Clinical and laboratory features in children with acute seizures

Of the 900 children with seizures, 427(47.5%) had single seizures and the remaining 473(52.3%) had 2 or more seizures. The median number of seizures in an individual child was 2 (IQR 1–3). The median age of children admitted with seizures was higher than that of children without seizures and fewer children with seizures were severely wasted. There were no differences in gender (table 1).

**Table 1:** Clinical and laboratory features on admission

**Patient characteristics**	**Patients with seizures**	**Patients without seizures**	**Crude OR, (95% CI)**	**P value**
Median (IQR) age, months	25.1 (12.5, 41.4)	13.1 (3.7, 36.3)	-	<0.001
Males, (%)	488/900 (54.2)	2,311/4,020 (57.5)	0.88 (0.76, 1.01)	0.074
Median (IQR) duration of illness, days	2 (1 – 3)	3 (2 – 4)	-	<0.001
Fever, (%)	829/900 (92.1)	2,649/4,021 (65.9)	6.05 (4.69, 7.89)	<0.001
Severe wasting, (%)	12/900 (1.3)	257/4,019 (6.4)	0.20 (0.10, 0.35)	<0.001
Severe anaemia, (%)	56/900 (6.2)	291/4,021 (7.2)	0.85 (0.62, 1.15)	0.282
Mean (SD) WBC, × 10^3^/μl	13.8 (9.9)	14.0 (9.5)	-	0.612
Proportion with malaria parasitaemia, (%)	489/885 (55.3)	647/3,910 (16.6)	6.18 (5.27, 7.24)	<0.001
Geometric mean (95% CI) parasite density, × 10^3^/μl	34.9 (28.5, 42.6)	20.8 (16.9, 25.7)	-	<0.001
Hypoglycaemia, (%)	27/864 (3.1)	136/3,682 (3.7)	0.84 (0.53, 1.29)	0.418
Acidosis, (%)	141/401 (35.2)	652/1,665 (39.2)	0.84 (0.67, 1.06)	0.140
Moderate – severe hyponatraemia, (%)	14/438 (3.2)	41/2,064 (2.0)	1.63 (0.81, 3.08)	0.117
Hypokalaemia, (%)	16/438 (3.7)	243/2,061 (11.8)	0.28 (0.16, 0.48)	<0.001
Hyperkalaemia, (%)	37/438 (8.5)	239/2,061 (11.6)	0.70 (0.48, 1.02)	0.056
Impaired renal function, (%)	60/407 (14.7)	408/1,864 (21.9)	0.62 (0.45, 0.83)	0.001
Bacteraemia, (%)	23/882 (2.6)	225/3,720 (6.1)	0.42 (0.26, 0.64)	<0.001

A history of fever was more common in patients with seizures. The mean axillary temperature at admission was higher (38.2 [SD 1.3]°C) in children with seizures compared to patients without seizures (37.6 [SD 1.2]°C), *p *< 0.001. However, the median duration of fever was shorter (2 days) in patients with seizures compared to those without seizures (3 days), *p *< 0.001 (table 1).

The median white blood cell counts and the proportions with severe anaemia were similar in both groups of patients. A total of 4,546 children had blood glucose levels determined at admission. Overall, hypoglycaemia was observed in 163 (3.6%) patients. The proportions of patients with hypoglycaemia were similar in both groups (table 1). Of the 2,066 patients with life threatening-features who had acid base status measured, 793(38.4%) had metabolic acidosis. The proportions of patients with metabolic acidosis admitted with and without seizures were also similar. However, impaired renal function, hypokalaemia, and moderate-severe hyponatraemia were more common in children admitted without seizures (table 1).

#### Aetiology of acute seizures

Over 80% of the seizures were associated with an infectious illness (table 2). Malaria was the commonest aetiological factor associated with seizures and over 50% of the children admitted with acute seizures had malaria as the primary diagnosis. The number of children with seizures and a positive malaria slide was even higher: 489/885 (55.3%) of the children with seizures had positive malaria slides compared to 647/3,924 (16.5%) children without seizures, *p *< 0.001. The risk of seizures increased with the parasite density. Children who experienced two or more seizures had a higher geometric mean parasite density (34,167 [95%CI 25,910–45,054]/μL) compared to those with single or no seizures (23,962 [95%CI 20,135–28,517]/μL), *p *< 0.001.

**Table 2:** Aetiological diagnoses associated with acute seizures and outcome

**Diagnosis**	**Patients with seizures, (%, n = 900)**	**Patients without seizures, (%, n = 4,021)**	**Crude OR, (95% CI)**	**P value**
Malaria	479 (53.2)	482 (12.0)	8.35 (7.08, 9.85)	<0.001
Respiratory tract infections	138 (15.3)	956 (23.8)	0.58 (0.47, 0.71)	<0.001
Pyogenic meningitis	24 (2.7)	20 (0.5)	5.48 (2.89, 10.5)	<0.001
Encephalopathy of unknown cause (probable viral encephalitis)	15 (1.7)	12 (0.3)	5.66 (2.46, 13.3)	<0.001
Gastroenteritis	39 (4.3)	564 (14.0)	0.28 (0.19, 0.39)	<0.001
Severe malnutrition	13 (1.4)	230 (5.7)	0.24 (0.13, 0.42)	<0.001
Trauma	4 (0.4)	96 (2.4)	0.18 (0.05, 0.48)	<0.001
Poisoning	1 (0.1)	42 (1.0)	0.11 (0.003, 0.62)	0.007
Prematurity	1 (0.1)	82 (2.0)	0.05 (0.001, 0.31)	<0.001
Hypoxic ischaemic encephalopathy	5 (0.6)	98 (2.4)	0.23 (0.07, 0.55)	<0.001
Neonatal sepsis	33 (3.7)	406 (10.1)	0.34 (0.23, 0.49)	<0.001
Others	149 (16.6)	1,076 (26.8)	0.54 (0.45, 0.66)	<0.001
Outcome				
Died	28 (3.1)	226 (5.6)	0.54 (0.35, 0.81)	0.002
Neurological deficits	11 (1.3)	0 (0)	-	<0.001

Apart from being the commonest illness associated with seizures, malaria was also associated with the highest seizure frequency and status epilepticus. The median number of seizures in children with malaria was 2 [IQR 1–4] compared to 1 [IQR 1–3], (*p *= 0.004) in those with other illnesses. Out of the 98 children with status epilepticus, malaria was the primary diagnosis in 56 (57.1%). In addition, seizures in children with malaria had the longest duration.

Two hundred and forty eight children out of 4,602 (5.4%) had positive blood cultures but bacteraemia was more common among patients without seizures (table 1). *Streptococcus pneumoniae *was the commonest cause of bacteraemia in both groups of children but was more common among patients with seizures, (10/23 [43.5%] vs 31/225 [13.8%], p < 0.001). Other causes of bacteraemia in children with seizures included beta haemolytic Streptococci (3), *Haemophilus influenzae *type b (2), *Escherichia coli *(2), viridans Streptococci (2), non typhi Salmonella (2), *Staphylococcus aureus*(1) and Pseudomonas (1). Among patients without seizures bacteraemia was caused by *Staphylococcus aureus*, *Streptococcus pneumoniae, Haemophilus influenzae*, *Acinetobacter*, viridans Streptococci, non typhi Salmonella species and several other gram-negative rods. Lumbar puncture was performed on 594/900 (66%) children with seizures from within the study area and a diagnosis of meningitis made in 44 children. The causes of pyogenic meningitis included *Streptococcus pneumoniae *(9), *Haemophilus influenzae *type b (2), *Escherichia coli *(1) and non typhi salmonella (1).

The aetiology of seizures varied with age. Sepsis was the most important cause of neonatal seizures. Pyogenic meningitis, gastroenteritis and respiratory tract infections were the most common diagnoses in children 2–5 months while malaria was the most common illness in children 6 months or older (table 3). *Staphylococcus aureus, E. coli *and other gram-negative rods, viridians Streptococci,*Acinetobacter*, and *Streptococcus pneumoniae *were the organisms most commonly isolated in neonatal sepsis.

**Table 3:** Seizures manifestation, main aetiological factors and outcome by age group of children with seizures

**Description of seizures**	**Age group of patients**				
	
	**0 – 1 month, N = 43, (%)**	**2 – 5 months, N = 36, (%)**	**6 – 36 months, N = 535, (%)**	**37 – 72 months, N = 237 (%)**	**>72 months, N = 49, (%)**
Proportion with seizures	43/736 (5.8)	36/514 (7.0)	580/2,374 (22.5)	258/742 (31.9)	55/555 (8.8)
Parental description of seizure event					
Focal	13 (30.2)	7 (19.4)	72 (13.5)	21 (8.9)	8 (16.3)
Focal, secondarily generalised	6 (14.0)	0 (0)	25 (4.3)	7 (3.0)	0 (0)
Generalised	21 (48.8)	25 (69.4)	423 (79.2)	199 (84.0)	39 (79.6)
Not described	3 (7.0)	4 (11.1)	16 (3.0)	10 (4.2)	2 (4.1)
Number of seizures, median (IQR)	3 (2, 5)	1 (1, 3)	2 (1, 3)	2 (1, 3)	1 (1, 2)
Level of consciousness at admission					
Normal	31 (72.1)	26 (72.2)	401 (74.9)	171 (72.2)	33 (67.4)
Prostration or mild impairment	8 (18.6)	6 (16.7)	73 (13.6)	39 (16.5)	5 (10.2)
Agitation	3 (7.0)	3 (8.3)	8 (1.5)	3 (1.3)	3 (6.1)
Coma	1 (2.3)	1 (2.7)	61 (10.5)	29 (11.2)	8 (14.6)
Leading diagnoses associated with seizures by age group					
1	Neonatal sepsis 28 (65.1%)	Respiratory tract infections, 10 (27.8%)	Malaria, 303 (56.6%)	Malaria, 155 (65.4%)	Malaria, 18 (36.7%)
2	Pyogenic meningitis, 7 (16.3%)	Acute gastroenteritis, 6 (16.7%)	Respiratory tract infections, 85 (15.9%)	Respiratory tract infections, 37(15.6%)	Respiratory tract infections, 6 (12.2%)
3	Hypoxic ischaemic encephalopathy, 5 (11.6%)	Pyogenic meningitis, 6 (16.2%)	Acute gastroenteritis, 27 (5.1%)	Acute gastroenteritis, 4 (1.7%)	Others 21 (42.9)
Outcome					
Death	7 (16.3)	0 (0)	15 (2.8)	3 (1.3)	3 (6.1)
Sequelae	3 (7.0)	1 (2.8)	5 (0.9)	3 (1.3)	1 (2.0)

#### Immediate outcome of acute seizures

Children admitted with seizures had a shorter hospital stay (median 3 [IQR 2–4] days) compared to patients without seizures, (4 [IQR 2–6] days), *p *< 0.001. Mortality was also lower among patients with seizures compared to patients without seizures; 28/900(3.1%) children with seizures died compared to 225/4,021(5.4%) children without seizures. Of the 28 deaths of children with seizures, 11 died of malaria, four of pyogenic meningitis and four possibly had viral encephalitis. Another four children had neonatal sepsis, birth asphyxia or prematurity. Table 4 is a summary of the diagnoses in patients with poor outcomes. The three other deaths were that of a preterm baby with neonatal seizures, a child with hydrocephalus and an infected shunt and another child with sickle cell disease and overwhelming sepsis.

**Table 4:** Diagnosis and poor outcome in children with seizures

**Diagnosis**	**Mortality, (%)**	**Gross Neurological deficits on discharge, (%)**
Malaria		
Cerebral malaria	9/63 (14.3)	4/54 (7.4)
Non cerebral malaria seizures	2/416 (0.5)	1/414 (0.2)
Meningitis	4/24 (16.7)	1/20 (5.0)
Measles encephalitis	2/4 (50.0)	0/2 (0)
Encephalopathy of unknown causes	2/15 (13.3)	0/13 (0)
Neonatal infections	2/33 (6.1)	1/31 (3.2)
Ishemic hypoxic encephalopathy	2/5 (40.0)	2/3 (66.7)
Malnutrition	1/13 (7.7)	1/12 (8.3)
Gastroenteritis	1/39 (2.6)	0/38 (0)
Respiratory tract and ear infections	0/138 (0)	0/138 (0)
Other causes	3/150 (3.2)	1/147 (0.7)
Total	28/900 (3.1)	11/872 (1.3)

The children with seizures who died presented to hospital after a longer median duration of illness (3 IQR 1, 4 days) compared to survivors (2 IQR 1, 3 days, p < 0.001). Proportionally, mortality was highest in neonates, in patients admitted with coma, focal seizures or status epilepticus and among those with complications such as acidosis, hypoglycaemia, hyperkalaemia and impaired renal function, or with bacteraemia and pyogenic meningitis. The risk factors independently associated with death were coma, bacteraemia, hypoglycaemia and metabolic acidosis (table 5).

**Table 5:** Risk factors for death among children with seizures

**Risk factors for death**	**Deaths, (%)**	**Survivors, (%)**	**Crude Odds ratio (95% CI)**	**P-value**	**Adjusted odds ratio (95% CI)**	**P value**
Median (IQR) duration of illness, days	3 (1–4)	2 (1–3)	-	0.170	-	-
Median age (IQR), months	17(3–33)	25(13–42)	-	0.048	-	-
Focal seizures	8/25 (32.0)	149/839(17.8)	2.18(0.80, 5.45)	0.069	-	-
Status epilepticus	12/28 (42.9)	86/872 (9.9)	6.85(2.85, 16.0)	<0.001	-	-
Coma	14/28 (50.0)	73/872 (8.4)	11.0(4.62, 25.7)	<0.001	10.3(3.21, 33.1)	<0.001
Severe wasting	4/28 (14.3)	8/872 (0.9)	18.0(3.67, 72.3)	<0.001	-	-
Severe anaemia	4/28 (14.3)	52/872 (6.0)	2.63(0.64, 8.05)	0.073	-	-
Acidosis	16/20 (80.0)	125/381(32.8)	8.19(2.56, 34.2)	<0.001	7.19(1.94, 26.7)	0.003
Hypoglycaemia	6/27 (22.2)	21/837 (2.5)	11.1(3.30, 33.3)	<0.001	7.06(1.73, 28.7)	0.006
Hyperkalaemia	5/24 (20.8)	32/414 (7.7)	3.14(.86, 9.45)	0.025	-	-
Hypokalaemia	2/24 (8.3)	14/414 (3.4)	2.60(0.27, 12.4)	0.209	-	-
Hyponatraemia	2/24 (8.3)	12/414 (2.9)	3.05(0.31, 15.0)	0.141	-	-
Impaired renal function	14/24 (58.3)	46/383 (12.0)	10.3(3.94, 27.2)	<0.001	-	-
Bacteraemia	4/27 (14.8)	19/855 (2.2)	7.65(1.75, 25.6)	<0.001	16.9(3.39, 33.1)	0.001
Malaria	11/28 (39.3)	468/872(53.7)	0.56(0.23, 1.28)	0.133	-	-
Respiratory tract infections	0/28 (0)	138/872(16.4)	-	0.022	-	-
Gastroenteritis	1/28 (3.6)	38/872 (4.4)	0.81(0.02, 5.42)	0.841	-	-
Pyogenic meningitis	4/28 (14.3)	20/872 (2.3)	7.1(1.63, 23.5)	<0.001	-	-
Neonatal sepsis	2/28 (7.1)	31/872 (3.6)	2.08(0.23, 8.98)	0.320	-	-
Hypoxic ischaemic encephalopathy	2/28 (7.1)	3/872 (0.3)	22.3(1.77, 200)	<0.001	-	-

At discharge, gross neurological deficits were observed in 11/872 (1.3%) surviving children. These included motor deficits (spasticity and central hypotonia) in 7 children, speech and hearing difficulties each in 2 children and dystonia/athetosis in one child. Neurological sequela was independently associated with coma, status epilepticus, severe wasting and hypoxic iaschemic encephalopathy (table 6).

**Table 6:** Risk factors for neurological deficits on discharge

**Risk factors**	**Deficits on discharge, (%)**	**No deficits at discharge, (%)**	**Crude Odds ratio (95% CI)**	**P value**	**Adjusted odds ratio (95% CI)**	**P value**
Median (IQR) age, months	13 (0, 48)	26 (13, 41)	-	0.644	-	-
Focal seizures	1/11 (9.1)	148/828 (17.9)	0.46 (0.01, 3.28)	0.449	-	-
Status epilepticus	5/11 (45.5)	81/861 (9.4)	8.02 (1.88, 32.2)	<0.001	6.56 (1.05,44.9)	0.045
Coma	4/11 (36.4)	69/861 (8.0)	6.56 (1.37, 26.4)	0.001	18.4 (3.03, 112)	0.002
Severe wasting	1/11 (9.1)	7/861 (0.8)	12.2 (0.24, 110)	0.097	23.9 (2.15, 266)	0.010
Hypoglycaemia	1/11 (9.1)	20/826 (2.4)	4.03 (0.09, 30.8)	0.160	-	-
Malaria	5/11 (45.5)	463/861 (53.8)	0.72 (0.17, 2.84)	0.582	-	-
Bacteraemia	1/11 (9.1)	18/844 (2.1)	4.59 (0.10, 35.4)	0.120	-	-
Pyogenic meningitis	1/11 (9.1)	19/861 (2.2)	4.43 (0.10, 34.0)	0.130	-	-
Neonatal sepsis or tetanus	1/11 (9.1)	30/861 (3.5)	2.77 (0.06, 20.6)	0.318	-	-
Hypoxic ischaemic encephalopathy	2/11 (18.2)	1/861 (0.1)	191 (8.61,11278)	<0.001	158 (8.78,2827)	0.001

## Discussion

Children living in this rural area of Kenya experience a very high burden of acute seizure disorders. The incidence of acute seizures in children 0–13 years was 425 per 100,000/year and the incidence of non-epilepsy associated status epilepticus was 46 per 100,000 per year. Younger children were at a higher risk: the incidence of acute seizures in children younger than 5 years was 879/100,000/year and of that of non-epilepsy associated status epilepticus was 95 per 100,000/year. Malaria and neonatal sepsis were the major aetiological factors for acute seizures and malaria for status epilepticus. Mortality was 3.1% while 1.3% of the surviving children had gross neurological deficits at the time of discharge from hospital.

### The incidence of acute seizures

The incidence rate is age dependent and is highest in neonates in whom infections are the predominant cause. However, the peaks are seasonal and follow seasons of malaria transmission, which in turn is dictated by the rainfall pattern in the area. The numbers we describe excludes seizures in children with epilepsy and this incidence data is comparable to rates described in West Africa[9] except that the proportion of malaria related seizures is higher[19]. At least 11% of the children with acute seizures had status epilepticus.

This incidence data is an absolute minimum. It does not account for children from the study area that did not attend Kilifi district hospital as from previous studies, only about 20% of children who have seizures in the community are admitted to hospital (A Ngugi, personal communication) and that up to two thirds of deaths in children <5 years occur outside the hospital[25]. The map of incidence also suggests that distance from the hospital could have affected hospital attendance. Clearly, this suggests that our estimates could have greatly underestimated the incidence of acute seizures in children in this community. It may more closely represent the incidence of seizures in children in the study area that present for care rather the incidence of acute seizures in the region.

In a recent epidemiological study in the UK, the incidence of convulsive status epilepticus in children under the age of 16 years was estimated to be 18–20 per 100,000 per year[26], a rate similar to that in other developed countries[27]. Despite the fact that we excluded status epilepticus associated with the epilepsies, the incidence of status epilepticus in this setting was at least twice that described in the UK. The data clearly shows that malaria accounts for the majority of the excess cases.

### Aetiological factors associated with acute seizures in children

Infections were the predominant precipitants of acute seizures – over 80% of the seizures were associated with an infection. Malaria was not only the predominant infection in children 6 months and older but was also the main cause of status epilepticus. The median number of seizures in children infected with malaria was higher than that in children with other causes of seizures. The risk of seizures also increased with parasite density supporting the hypothesis that the parasite, *Plasmodium falciparum*, may be epileptogenic[28]. Sequestration of parasites in deep capillary beds in the cerebral microvasculature leading to local ischaemia/hypoxia may be responsible for this association (reviewed in[29]). Among incident cases, the association of falciparum malaria with cerebral sequestration also makes it difficult to distinguish purely febrile seizures from acute symptomatic seizures.

The main causes of acute seizures in children living in this setting (malaria and neonatal infections) and other important causes (pyogenic meningitis and hypoxic ischaemic encephalopathy) are illnesses that are preventable with available public health measures. Impregnated mosquito nets and other treated mosquito materials or residual in-door spraying are proven methods for malaria control[30]. Scaling up these measures can greatly reduce the number of children exposed to malaria related seizures. Immunization against *Haemophilus infleunzae *type b (Hib) has virtually stopped this organism from causing severe childhood illnesses in Western countries and significant progress has already been achieved with *Streptococcus pneumoniae*. Improved antenatal, perinatal (delivery by competent personnel and neonatal resuscitation programs) and postnatal care (cord care) can decrease the number of neonates exposed to seizures. Already, there is evidence to suggest that some of these programs are working in sub-Saharan Africa; in Kenya, there has been a drastic decline in the number of children with *Haemophilus infleunzae type b *infections after introduction of the pentavalent (DTP-HBV-Hib) vaccine[31] and in Eritrea, the incidence rate of malaria declined by over 80% after the introduction of multiple vector control methods, case management and surveillance [32].

Infection with HIV may potentially affect the clinical presentation of children with seizures. Since November 2006 (the last month of the study period), all children admitted to the hospital were systematically tested for HIV. The results show that 8% were HIV infected. However, since our initial study protocol didn't include systematic HIV testing, we are unable to assess the effect of HIV on seizure presentation. We acknowledge this as a limitation of the study.

### Outcome of acute seizures

Overall, mortality in children with seizures was low except in patients admitted with bacteraemia and neonatal infections, or those with complications such as hypoglycaemia, severe acidosis and coma. Unlike patients with malaria, pyogenic meningitis, neonatal infections or hypoxic ischaemic encephalopathy, almost all those with respiratory tract infections had single seizures and none died suggesting that these were probably febrile seizures. These febrile seizures may have contributed to the shorter duration of hospital stay among patients with seizures overall since in previous studies, acute symptomatic seizures were associated with longer periods of hospitalisation [28] and increased costs of care[33] compared to children without CNS involvement. Put together, these findings suggest that the poor outcome of seizures in tropical settings[12] is related either to the severity of the illness associated with the seizures or the presence of complications such as coma, hypoglycaemia, acidosis and status epilepticus.

In this community, annually, at least 12 children <5 years per 100,000 develop gross neurological impairments following exposure to acute seizures. Status epilepticus is a prominent risk factor and malaria is the main cause of status epilepticus. The association between infection with falciparum malaria and repeated or prolonged seizures may therefore be a major risk factor for neurological impairments in children in malaria endemic Africa. Other risk factors include a background of severe malnutrition and hypoxic ischaemic encephalopathy.

## Conclusion

In conclusion, children living in this rural malaria endemic area of Kenya have a high incidence of acute seizure disorders. The main causes are diseases that are preventable with available public health programs. Poor outcomes are associated with severe infection, concurrent biochemical complications, coma, status epilepticus and malnutrition.

## Competing interests

The authors declare that they have no competing interests. The Wellcome Trust played no role in the design and conduct of the study, the analysis and interpretation of data or the preparation, review and approval of the manuscript.

## Authors' contributions

RI designed the study, collected and analyzed the data and wrote the first draft. SG collected the data, provided clinical care for the patients and participated in data interpretation. TK, MK and HK participated in the design of the study, in data collection and in statistical analysis. MN, KM, BN and PK participated in the design of the study and interpretation of the data. CN participated in the design of the study, data collection, analysis and interpretation. All authors critically reviewed the manuscript.

## Pre-publication history

The pre-publication history for this paper can be accessed here:


